# Utilizing the physical green care environment to support activities of daily living for nursing home residents: a focused ethnographic case study

**DOI:** 10.1186/s12912-024-01782-7

**Published:** 2024-03-05

**Authors:** Svenja Cremer, Katharina Rosteius, Sandra M.G. Zwakhalen, H. Verbeek, Michel H.C. Bleijlevens, Bram de Boer

**Affiliations:** 1 Department of Health Services Research, Care and Public Health Research Institute (CAPHRI), Maastricht University, Duboisdomein 30, 6229 GT Maastricht Postbus 616, 6200 MD Maastricht, The Netherlands; 2 Living Lab in Ageing and Long-Term Care, Maastricht, The Netherlands

**Keywords:** Activities of Daily Living, Physical Care Environment, Green Care

## Abstract

**Background:**

The nursing home residents’ ability to carry out Activities of Daily Living (ADLs) is influenced by the physical care environment. One emerging area of interest in scientific research is the green care environment within nursing home care, where agricultural activities such as gardening and animal care are integrated alongside daily care. Previous research has neglected to explore how these environments can be employed to enhance ADL performance. This study, therefore, explores how a green care environment, specifically one with an animal shelter, can be used to support nursing home residents in their ADLs.

**Methods:**

A focused ethnographic case study was conducted in one nursing home. Data was collected employing participatory observations, informal conversations, and semi-structured interviews, which we analyzed by employing a thematic analysis.

**Results:**

Overall, 25 residents were observed for a total time of 89h, and interviews were conducted with 10 staff members. The nursing home integrates activities in the green care environment into daily care for a broad scope of residents. The analysis revealed four themes: (1) The (in)visibility of ADL, (2) Reciprocal care dynamics: Fostering ADL performance through connection and teamwork, (3) Seized and missed opportunities for meaningful integration of ADL in the physical green care environment, and Theme (4) Professional fulfillment and ADL task obligation: Views from staff and management.

**Conclusions:**

This physical green care environment carries the potential to enhance the residents’ daily activities and foster better staff-resident relationships. Yet, there are varying views among staff and management regarding its integration into the residents’ lives and care.

**Supplementary Information:**

The online version contains supplementary material available at 10.1186/s12912-024-01782-7.

## Background

Due to the progression of their disease, residents with dementia and related diseases increasingly depend on their environment when performing activities of daily living (ADL) [1, 2]. ADLs collectively refer to essential skills necessary for self-care and independence, encompassing activities like eating, bathing, and mobility which has first been described as a concept by Katz, Ford [3]. With nursing professionals supporting a person’s ability to perform ADLs, they lie conceptually at the heart of the nursing profession as the fundamentals of care framework illustrates [4]. As part of fundamental nursing, ADL care has received increased scholarly attention in the past decades focusing on the challenges and pre-conceptions [5, 6] as well as its importance for those who receive care [7]. Hence, it is not surprising that the World Health Organization (WHO) prioritizes the maintenance of underlying abilities to perform these activities, as essential to healthy aging [8].

The extent to which people are or are made capable of healthy aging depends in part on the environment they inhabit [9]. The physical environment should be carefully considered to assist in meeting a person’s needs and optimizing care routines [10–12]. The physical environment in dementia care encompasses various elements, such as unit size, residential ambiance, sensory stimuli, dining spaces, resident rooms, bathing and toilet facilities, and outdoor areas. These factors collectively contribute to the overall care environment for individuals with dementia [13]. Within nursing homes, the physical care environment can significantly impact the health and behaviors of residents [14] but its promising potential has not been sufficiently recognized within the scientific literature yet [15]. Inside spaces include, for example, the bathroom layout, orientation cues, more homelike character or noise, and light adaptations to the residents’ needs [15]. The outside environment and its natural elements including gardens, plants, and animals are often underestimated and overlooked opportunities for improving resident outcomes. For nursing home residents, access to the outdoors may be entirely restricted, and opportunities for outdoor experiences may be solely determined by facility personnel [16]. A care environment receiving recent scholarly attention especially in Europe is the green care environment [17] in which nursing home care offers agricultural activities (e.g. gardening and animal care) combined with care for people with dementia [18]. In the Netherlands, for example, nursing homes increasingly aim to integrate natural elements into daily care practices, recognizing the value of the presence of nature and animals and the activities associated with them [19].

As demonstrated by a recent review of Speckemeier, Niemann [20], innovative changes in the living environment such as smaller scales or opportunities for involvement in meaningful activities might be reasons why residents with dementia could better maintain their abilities in ADL functioning. Specifically for the outdoor environment a different review indicated positive effects on mental health, physical activity, structure, and meaningfulness in residents being involved in activities around animals and plants [21]. Research has shown that environments including activities with animals contribute to a general increase in ADL performance in, for example, stroke survivors [22] as well as to ADL-related outcomes such as food and fluid intake in community-dwelling older people with dementia [23]. These findings hold particular importance for nursing professionals as they play a crucial role in providing ADL nursing care for residents in nursing homes [7]. However, it remains unclear whether and how this environment is used to facilitate ADL performance.

It appears that the purposeful use and integration of the physical green care environment in ADL care remains challenging as up until now they mainly serve recreational purposes [19]. It remains unclear how the physical green care environment can be used to facilitate ADL performance. In fact, a review of Woodbridge, Sullivan [24] emphasizes the gap in the literature as to how the environment can support ADLs in the living environment of older people with dementia. They furthermore emphasize the need to enhance insights into the interactions between older persons with dementia and their surroundings while integrating their perspectives. Therefore, this study aims to answer the following research question: How can a physical green care environment be used to facilitate ADL performance in residents of a nursing home?

## Methods

A focused ethnographic case study was conducted [25] adhering to the Standard for Reporting Qualitative Research (SRQR) [26]. Aligned with the exploratory nature of our research, ethnography presented a suitable approach as it allows an immersion into real-life situations to identify patterns, relationships, and meanings within the entire environmental context [27]. This approach was chosen in line with this study’s aim since it allows the researchers to get insights into the living world of how this environment is used to facilitate ADL functioning in this particular setting. It not only permits the observation of residents and staff behavior within the environment during activities but also facilitates the observation of interactions and relational aspects as they unfold. A focused ethnography, as opposed to prolonged immersion, employs concentrated data gathering to investigate a specific topic. In health services research, this approach proves beneficial for rapidly gaining a thorough understanding of a particular topic involving short-term and targeted data collection [25].

### Setting

Despite Dutch policies that encourage individuals to reside in their own homes as long as possible, nursing homes in the Netherlands are primarily an option for the most vulnerable individuals in society, such as people living with dementia [28]. In 2017, 38% of the people living with dementia resided in Dutch nursing homes. The case for this study was a nursing home (*n*∼200 residents) for residents with psychogeriatric diagnoses including early-onset dementia, other forms of dementia, Korsakoff’s and, Parkinson’s disease. All residents living in the nursing home were in need of 24-hour care. However, depending on the residents’ needs and diagnosis, ADL care needs varied greatly. This nursing home combines large- and small-scale living, ranging from 11 to 24 residents per ward. To meet the different interests of the residents, the nursing home offers different activities for residents including carpeting, painting, musical activities and swimming or other physical exercise. Additional descriptions of the setting and its physical green care elements are described in the results section.

### Sampling

For the observations, we used a convenience sample of residents and nursing, activity staff, and managers, based on their presence during the observed activities with and around animals (Table 1). Moreover, we selected ward managers who were responsible for the residents we observed. The selection of staff members for interviews strived towards selecting a variety of professionals including Registered Nurses (RNs), Certified Nursing Assistants (CNAs), activity staff, and ward managers with different roles and experiences in using the environment including animals.

**Table 1:** Characteristics of nursing home wards, the resident’s main diagnosis and the frequency of participation in the outside environment

Ward	Residents participating/Residents living in the ward*	Residents’ main diagnosis	Weekly frequency of organized activities per ward (total time spent per week)
A	8/24	Geriatric psychiatry	1x per week (1.5 h)
B	2/11	Geriatric psychiatry	2x per week (1 h)
C	1/27	Korsakoff’s disease	1x per week (1 h)
D	5/20	Early onset dementia (early and advanced stages)	5x per week (3 h)
E	0/20	Parkinson’s disease	3x per week (4.5 h)
F	5/15	Advanced dementia	2x per week (1.5 h)
G	2/14	Korsakoff’s disease	8x per week (7 h)
H	2/14	Korsakoff’s disease	6x per week (7.5 h)

* The number of residents living in the ward is not equal to the number participating in the activities

Out of the 28 residents who took part, three legal representatives of those involved in the outdoor environment opted not to provide consent for participation in this study

### Data collection

All data, including participatory observations, informal conversations, and interviews, were collected from January 2022 to September 2022.

### Resident and staff characteristics

We used a short questionnaire to collect general resident demographics (name, age in years, main diagnoses as reported in the electronic resident records, and name and type of ward they live in). Data was collected by the social worker of the care organization who had access to the electronic resident records. During the interviews, staff, data was collected including name, age, profession, and the ward they work on.

### Participatory observations and informal conversations

To explore the interplay between the physical green care environment and the performance of ADLs of residents, participatory observations were conducted. This allowed the researchers to immerse in the setting and engage with the residents and staff [29]. The unit of observations centered around the activities taking place in the physical green care environment.

Observations took place before, during, and after the scheduled activities. This meant that researchers accompanied residents from their ward to the outside environment. Following this, the planned activities at the animal shelter took place. Afterward, the researchers, along with the staff, escorted the residents back to their ward, concluding the session. The approach involved open and flexible observations to capture the natural flow of activities and interactions, providing a more authentic and contextually rich understanding [30].

When conducting the observations we took a stepwise approach of descriptive, focused and selective observations inspired by Spradley [31] and Whitehead [32] in which we gradually added structure as we moved further along in this iterative process.

In the descriptive phase we first entered the field aiming to “naturally inquire” as much information as possible on the context guided by questions such as *what* is happening as well as *who, where*, *when* and *why*. In line with our research questions, this also meant that we paid particular attention to element of the physical environment including space and objects To pay particular attention to the environment we devoted our observations to the green care environment, such as the spatial layout, objects, animals, distances between the outside environment and wards. In this phase, the researcher’s participation within the activities was limited accompanying residents and staff to the activities and being there. Hence, we also got a general impression of the kind of activities taking place in that environment (including ADLs) as well as the residents and staff members participating in these.

In the focused part of our observations, we moved from general observations to exploring specific behaviors of and interactions between residents, staff, and environment within the activity context. This meant gradually increasing our participation to experience first hands how for example staff prompts, or animals stimulate ADL performance. Leading for the selection of observation moments were six predefined ADL categories based on the Barthel index which assesses ADLs including washing mobility (un)-dressing grooming toileting, and eating and drinking) [33]. This means that the researchers were especially mindful of events related to these ADL categories during the activities.

In the selective phase of our observations we were looking at patterns of interactions, their meaning as well as the goals and motivations of those involved in the activities. Throughout this phase, as active participants, we used naturally occurring informal conversations to understand how participants attributed meaning to the activities and their environmental context. In case of residents not being able to engage in conversations, we specifically focused on the non-verbal reaction and behavior of residents.

The first author performed most of the observations; the second author joined in one-third of the observations for purposes of mutual reflection and additional perspectives. After each observation, the authors briefly ‘jotted’ or sketched a record of the observed events in keywords into a journal they kept with them at all times. These were then processed into extensive and descriptive field notes as described by Emerson, Fretz [27].

For illustrative purposes, photos of some residents engaging in activities were taken with consent for publication from the residents or their formal representatives. Participatory observations took place in January and February 2022. Overall, we observed the residents for a total time of 89 h.

### Semi-structured interviews

The perspectives of nursing and activity staff as well as ward managers were explored in semi-structured interviews, enabling them to share their views, attitudes, interpretations, and opinions on the use of this environment. A topic guide was developed to guide the interviews which included questions targeted to the role of the participant, his or her experiences and, perceptions of the green care environment and its use. In relation to ADL, questions about the purpose of using this environment were asked, followed by questions on what this environment in return means for the daily life and ADLs of the residents. Questions were openly formulated leaving room for what the participants deemed important on how the environment is used to facilitate ADL. For instance, what is your view on the activities that take place in the green care environment? To what extent does the environment and the activities relate to daily care? Do you see a connection between the activities in the green care environment and the daily activities of the residents (washing, dressing, eating, drinking, mobility)? Moreover, we used examples of the observations to illustrate situations and to gather the interviewee’s in-depth perspectives on these examples.

We conducted two pilot interviews to get acquainted with the guide and adapt it where necessary. The interviews were conducted between June and September 2022. In total, ten interviews were conducted, which on average lasted 31 min ranging from 22 to 40 minutes.

### Analysis

We inductively used our data using the thematic analytical approach by Clarke, Braun [34]. We were furthermore guided by their 15-point checklist of criteria for good thematic analysis to increase the dependability of the results and maximize rigor [31]. As a tool for coding our data, we used MAXQDA 2022 (VERBI Software, 2021). In our analysis, we used a stepwise approach was used starting with the field notes which were read in depth and given a preliminary initial open coding layer. As examples from the field notes were used in the interviews, this step was necessary to get familiar with the data and to form an initial impression and distill illustrative examples for deeper insights.

As a second step, we indicatively coded the interviews as well by generating initial open codes to the interview transcripts. We methodically examined the complete dataset, dedicating thorough attention to elements relevant to the research question. Once field notes and interviews were foreseen of an initial coding layer we proceeded to the third phase in which we shifted our focus to generating themes. We began by merging and matching codes to bringing together all the important data extracts to identify overarching themes. As we understood relations between overarching themes, we concluded this phase with a set of potential themes and sub-themes, along with all the coded data extracts related to them. In the final phase of analysis, we reviewed and refined our themes by reviewing all codes based on their coherence and meaningfulness to the generated theme as well as judging whether the theme itself adequately represents the coded data. This included that at some instances we moved segments to other (sub) themes or new sub-themes were created until we were satisfied that themes adequately captured the contours of the coded data. Finally, we looked at the accuracy of our individual themes in relation to the data set as a whole and adjusted where necessary. We added a codetree describing our themes, subthemes, and examples of codes to Appendix 1.

To ensure accuracy in interpreting the data, a combination of consensus coding and split coding [35] was used. The same two pages of field notes and two interviews were openly coded by the first two authors, and the results were compared on a one-to-one basis. Once consensus was achieved on the initial data, the remaining data was divided equally between the first two authors to streamline the process. Furthermore, weekly meetings were held by the first two authors to continually compare new data with previously coded information. The codes and themes were collectively discussed by the research team in monthly meetings.

Attaining data saturation in ethnographic research can pose challenges, given the extensive data collected throughout the limited study period [36]. Moreover, the concept of data saturation has encountered increased criticism in qualitative research due to its inherent vagueness [37]. Consequently, the focus of this study was directed towards obtaining rich, contextualized data for the research setting.

### Reflexivity

Reflexivity was increased by the first two authors, who kept reflective notes, before and after data collection, on their own preferences and pre-conceptions. Especially rapid ethnography reflexivity can enhance team relationships and the caliber of the research output [38]. All members of the research team have a background in nursing home care with additional backgrounds in occupational therapy (SC), health economics (KR), nursing (SZ), psychology (HV, BdB), and physiotherapy (MB). Moreover, a part of the research team holds expertise in innovative care environments for persons living with dementia, for example, Green Care Farms (KR, HV, BdB). It is precisely this composition of backgrounds that has ensured an examination of identical data from various perspectives, identifying variations in interpretations through discussions. Regular research team meetings increased mutual reflection on the research background and previous work in clinical practice as well as own pre-conceptions on the use of the physical environment and affinity with animals and nature.

Looking at how the cultural background of researchers could have influenced the results, we consider the influence of language, and geographical region minimal since researchers and participants lived in a similar geographical region in the south of the Netherlands and or Germany. Regarding cultural values linked to the research question, we were aware of differing values of good care. For example, traditional care approaches might value safety and taking over activities over stimulating independence. However, since regular reflection on potential cultural influences was incorporated in the research meetings we consider this influence on our results minimal.

### Ethics

Ethical approval was gained from the Research Ethics Committee (approval number: METCZ20210138). In order to conduct the observations, we obtained written informed consent from the legal representatives of the residents as the residents themselves were unable to provide formal consent due to cognitive limitations. For the interviews, the participating staff members signed informed consent during the interview. To prevent ethical issues for nursing home staff during observations, the presence of the researchers during the activities was communicated by mail within the nursing home, and the researchers introduced themselves and the studies’ aim to the staff members In addition, residents were always treated with respect and dignity by having the observers being integrated into the social context as much as possible.

## Results

The results section consists of three parts: (1) characteristics of the participants, (2) a consideration of the setting, and (3) identified themes from the thematic data analysis.

### Participants

Characteristics of participating residents who were mostly males are displayed in Table 2. These characteristics describe the variety in both diagnosis and age.

**Table 2:** Characteristics of participating residents

	Residents’ main diagnosis in the wards	Residents participating	Mean age [range]	Gender (Male %)
Ward A	Geriatric psychiatric diagnoses*	8	80.1 [73–92]	33
Ward B	Geriatric psychiatry diagnoses*	2	57.5 [53–62]	100
Ward C	Korsakoff’s disease	1	76	0
Ward D	Early-onset dementia (early and advanced stages)	5	57.2 [50–68]	80
Ward F	Advanced dementia	5	80.6 [75–91]	40
Ward G	Korsakoff’s disease	2	61	100
Ward H	Korsakoff’s disease	2	65 [68–74]	100
Total		25	68,2 [50–91]	57

* Common diagnoses found in these wards included schizophrenia, intellectual disabilities, various types of dementia, or bipolar disorder

Staff characteristics are displayed in Table 3.

**Table 3:** Characteristics of care staff participating in interviews (*n* = 10)

Participant *	Age	Gender	Position	Ward	How their position relates to the environment and activities
**Nursing professionals**					
Gabrielle	51	Female	Nurse assistant	F	Responsible for meal-time care
John	28	Male	Certified Nursing Assistant	D	Responsible CNA for two residents participating in activities
Liza	47	Female	Certified Nursing Assistant	G	Responsible CNA for two residents participating in the activities
Rose	38	Female	Registered Nurse	F	Delivery and coordination of nursing care and identifying the residents’ preferences
**Activity staff**					
Emma	44	Female	Activity staff	D	Cares for residents with advanced dementia using the environment
Jennifer	24	Female	Activity staff	F	Cares for residents with advanced dementia using the environment, participates in activities
**Other professions**					
Jess	27	Female	Social worker	-	Responsible for managing and coordinating the environment and activities with and around animals
**Managers**					
Ava	49	Female	Ward manager	A, F	Responsible for care delivered in wards
Monica	63	Female	Ward manager	D	Responsible for care delivered in wards’ coordination volunteers and central coordination of activities throughout the nursing home
Shelly	59	Female	Ward manager	G, H	Responsible for care delivered in wards and the creative workshop

* Pseudonyms

### Considering the setting

#### A description of the residents’ ADL care needs and context

Where residents with Korsakoff’s disease were often younger and able to perform their ADLs with prompts and structural support, other residents, for instance, with severe dementia, fully depend on support in ADL. During the observations, differences in mobility among the 25 observed residents were noted. Where most residents were able to walk independently (*n* = 11), a significant proportion used either a walker (*n* = 4) or a wheelchair (*n* = 8) or depended on physical assistance (*n* = 2). Residents using a wheelchair were not able to use it themselves and depended on staff to be mobile. Depending on the ward, the residents used a shared bathroom. A toilet was present in each resident’s single room. In each ward, residents share a dining- and living room, and a kitchen where resident share meals with and without support. Additionally, the facility includes a restaurant open for residents, staff and visitors. Staff members regularly visited with residents after the schedules activities outside.

Residents were allowed to move around inside the nursing home. To access the outside environment, residents depended on staff. Some residents were in possession of a key that opened the doors to the outside.

#### The physical green care environment

**Fig. 1:**
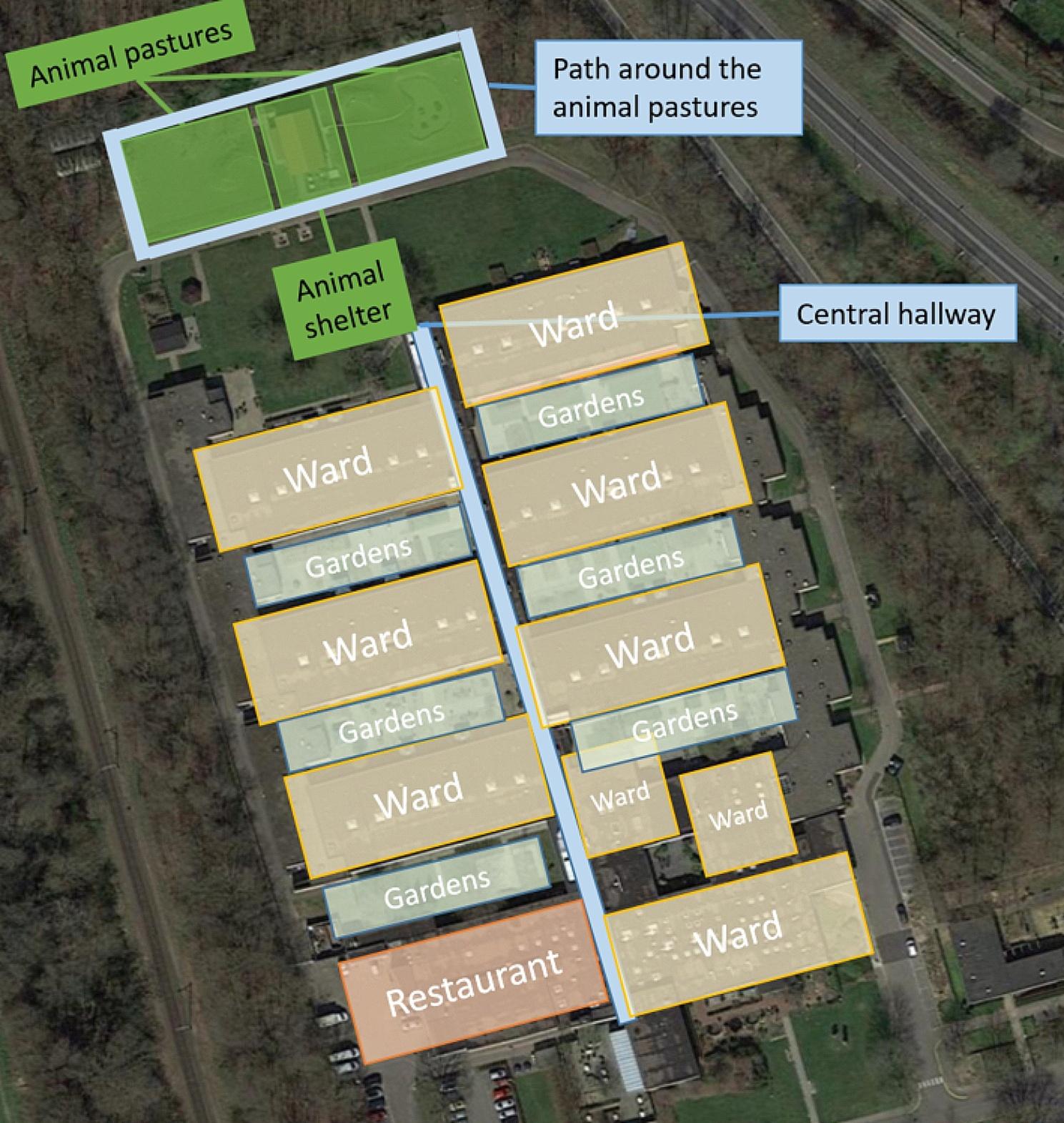
Layout of the nursing home and its green care environment Adapted from Map data ©2023 Google

**Fig. 2:**
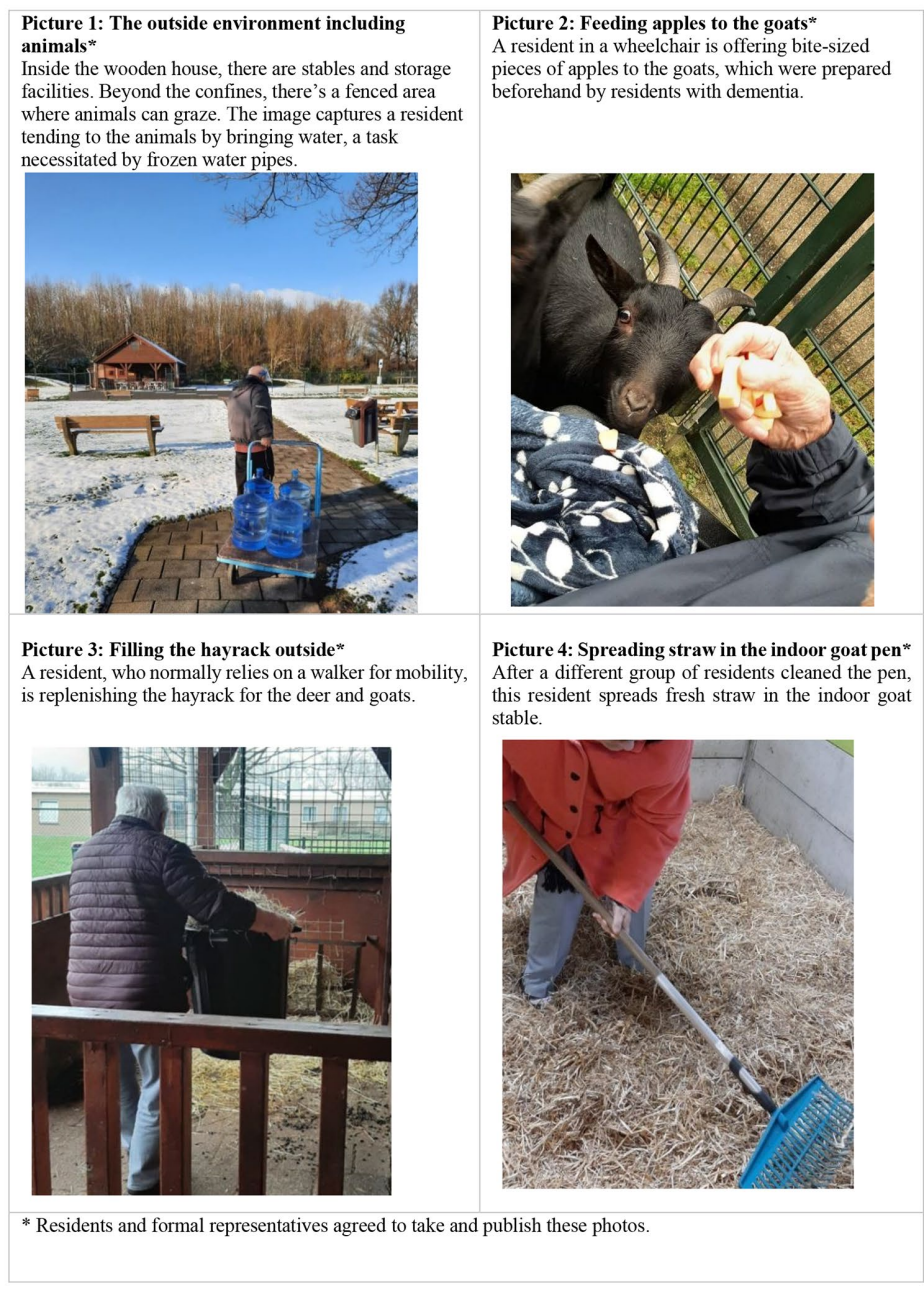
Impressions of the green care environment and its use

The nursing home was entirely situated at ground floor level. Each ward had access to a small garden area where some residents grew flowers or vegetables. Additionally, a large park was shared by the entire nursing home. Here, an animal shelter was built a few years ago. The animals present included deer (*n* = 4), goats (*n* = 2), chickens (*n* = 12), and geese (*n* = 2). The animals lived in a fenced area of ca. 1600m^2^ behind the nursing home to be reached by a paved path of ca. 80 m (Fig. 1). At the heart of this space stands a wooden house, housing stables for chickens and goats, as well as storage for their feed. Encircling the fenced area, a path beckons residents, staff, and visitors for a leisurely stroll. Beyond the animal enclosure lies a gated forest. The wooden house and stable are also secured, with select employees and residents having access via a key. Figure 2 provides a visual representation of the physical components of this green care environment and its associated activities. With the goal of integrating the green care environment into the residents’ daily routines, a variety of activities centered around the animal shelter was planned for each ward. An illustrative example of how the activities are planned is provided in Table 4. These activities were tailored to the specific ward, taking into account the residents’ preferences and abilities, and included tasks such as visiting and interacting with the animals or helping with stable maintenance. The planning and execution of these activities were overseen by a social worker, with the assistance of activity staff and nursing professionals from the respective wards. The social worker was also responsible for the health and safety of the animals including vet appointments and the collaboration with local animal welfare authorities.

**Table 4:** Example of scheduled activities with and around animals

Time	Activity
8:30–09:00	Residents living with early-onset dementia feed the goats in their inside stable and letting them outside to join the deer in grazing. Then residents feed hay to the deer outside, clean the water buckets, and refill them. Residents check for eggs in the chicken coop and collect them, often taking them to their ward for breakfast.
10:30–12:00	People living with advanced dementia engage in a ‘Cuddle activity’. They first take a walk to the animals and have a drink there. Residents prepare fresh food for the animals (apples, carrots) and feed them. Some residents engage in ‘farm-like’ activities based on their interests and use the broom to clean the premise.
13:30–15:00	Residents with Korsakoff’s disease clean the inside pens for chicken and goats. Exchanging straw, hay and cleaning the floor with water. Carrying the dirty straw outside with a wheelbarrow (ca. 4 wheelbarrows). Cleaning the outside premise, sweeping the deer and goat manure, collecting the dirt in a wheelbarrow, and emptying the wheelbarrow in a container 85 m away from the stables.
15:00–16:30	Residents with Korsakoff’s disease care for plants outside the animal premise, or doing construction work (e.g. fences, building hotels for insects)
16:30–17:00	Residents living with early-onset dementia bring the goats to their inside stables and feeding them. Checking on all animals before nighttime.

### Themes identified based on thematic data analysis

The thematic data analysis revealed four themes: (1) The (in)visibility of ADL, (2) Reciprocal care dynamics: Fostering ADL performance through connection and teamwork, (3) Seized and missed opportunities for meaningful integration of ADL in the physical green care environment, and (4) Professional fulfillment and ADL task obligation: Views from staff and management.

#### Theme 1: The (in)visibility of ADL

The theme (in)visibility of ADL is characterized by the tension of ADLs being visible to observers as an integral part of the organized activity within the green care environment. Subthemes include the ‘*Visibility of ADL and before, during and after activities*’ as well as ‘*Invisible aspects of ADL within and beyond activities*’.

The overall theme highlighted a contrast into ADLs were visible in the context of the physical environment. The subtheme ‘*Visibility of ADL and before, during and after activities*’ shows how ADLs were quite noticeable prior, during, and before and after activities.

The initial theme highlighted a contrast in how ADLs were visible in the context of the physical environment. ADLs were quite noticeable prior, during, and after activities. For example, prior to the activity when residents get dressed appropriately according to the weather, putting on jackets and suitable footwear. This resulted in additional ADL care moments for those residents participating in activities. Residents diagnosed with Korsakoff’s disease, who participated in the stable cleaning, were provided with specialized work clothing, which mandated a full change before engaging in the tasks. It also became evident that the frequency and scheduling of activities such as shower times were adjusted to align with the scheduled activities in the green care environment.

The findings showed how perceived benefits of the green care environment use appeared to extend themselves to periods before or after activities. In the context of ADL morning care, one of the staff members even mentioned how the animals seemed to motivate some residents to get up in the morning:

*“They always get up for the animals in the morning […]. I’ve rarely experienced residents not going. Whereas to activities like the carpenter workshop or choir, they often say, ‘No, I’m not coming.’ […]. Of course, the animals also need to eat and that’s also important and I think they have that in the back of their minds, I mean of course, the animals will still get food if they don’t go with them, but I still think it’s a feeling inside and they also just like it.*” [John, CNA, l.115–118].

It was observed that residents with dementia are more able to voice their ADL needs after activities, as noted by a ward manager:

*“I just saw these residents coming back from visiting the animals. And when I normally ask her [a resident] something, there’s no response. Now she can indicate to me that she is thirsty after visiting the animals. And then I find it really special that she can indicate to me that she is thirsty.”* [Monica, Ward manager, l.287–290].

During the activities, the green care environment also encouraged residents to prepare and eat food such as apples, drink tea or coffee, and to engage in more demanding, mobility-related ADLs. Residents who were able to walk covered significant distances during the activities, including walks from the ward to the animal shelter and back, as well as engaging in physical activity when for example cleaning the stables, getting hay, and emptying the wheelbarrow. One of the residents was very aware of the physical benefits of helping in the animal shelter and even applied advice from his physiotherapist:

*“My physiotherapist always says how I need to avoid rotating movements because of my hip. This is why I clean the stables like this [resident moves around using small steps].”* [Fieldnote extract].

After the activities, ADLs such as washing hands or undressing were observed regularly. For example, residents were encouraged to wash their hands or to clean their shoes using a built-in shoe brush before re-entering the building. Depending on the work the residents did, they were encouraged to shower afterwards.

Invisible aspects of ADL within and beyond activities.

Regardless of the number and clear presence of ADLs, the topic seemed less visible when talking to staff members. Although some staff members perceived the use of the green care environment as beneficial in terms of prevention of physical decline or being overweight, most described it solely as a valuable asset for residents to have meaningful activities, a work-life structure, or moments of relaxation. The researchers observed moments where some staff members used the animal shelter to eat together with residents and carried food and drinks to eat on a terrace in front of the stable.

*“While sitting on a bench a staff member arrives on a ‘duo-bike’ where she and a female resident can ride the bike next to each other. We engage in small talk on the weather and when I ask what she will be up to, she explains how she took some sandwiches for the resident to eat while watching the animals. According to her, the resident will eat more when looking at the animals. Inside the ward, they struggle to achieve a sufficient food intake for this resident. That’s why they sometimes have lunch outside near the animals.”* [Fieldnote extract].

Nevertheless, in the majority of cases, the perceived aim and objective of utilizing the environment were identified as ‘being outdoors,‘, ‘a sense of purpose and meaning’, ‘alleviating agitation,’ or ‘providing structure’ ‘social connectedness’ or ‘intentionally engaging residents’. Enhancing ADLs was not generally recognized as one of the purposes of utilizing the green care environment.

#### Theme 2: Reciprocal care dynamics: fostering ADL performance through connection and teamwork

Theme 2 is illustrates the social and relational component of the use of the green care environment especially between residents and staff. It is defined by the subthemes ‘*Strengthening the care relationship within and beyond green care activities’* and *‘Reciprocity through equality and expertise*.

As part of the subtheme ‘*Strengthening the care relationship within and beyond green care activities*’, staff described how using the environment gives them an easier entry point to have conversations with the residents about their day and their interests. This conversation starter made it easier for residents to share their concerns and preferences, which in the experience of staff members, strengthened the care relationship. In some cases, the use of the green care environment built a relationship, which had a direct influence on ADL morning care. An activity staff member described how the use of the environment allowed her to support a resident with dementia and complex care needs in ADL care activities whereas the resident refused care from other nursing professionals:

*“This lady refused all activities and care […] she is very distrustful of everything and everyone and you then have to work towards it very slowly and try to build a bond and little by little I was able to go to the animal shelter. In the beginning, she went along grumbling reluctantly, but from the first moment she has been in there she brightens up and talks to the animals […] the look becomes milder in the face […] and the eyes start to shine, she starts to talk to the animals she starts to pet the animals…[…]. And so I found an entrance to be able to take care of her and shower her. Each time step by step and after showering we went to the animals together and at some point, she started linking that so every time I went to groom her, she asked if we were going to see the deer again.”* [Emma, activity staff, l.52–71].

The green care environment allowed for shared positive moments where there is room for humor and jokes on the one hand but also reactions of residents who enjoy being outside and around animals. In the observed activities, staff members took the time to wrap up activities by spending time together outside or in the restaurant while talking, smoking a cigarette, or enjoying a cup of coffee. In interviews, staff members explained that experiencing these moments helped them to foster a relationship beyond the intimate care environments or situations in which residents were expected to perform or behave in a certain way.

As a result, the subtheme *‘Reciprocity through equality and expertise’* highlights how using the green care environment and caring for animals together reduced hierarchical structures during the activities since both parties care for a third party, the animals. Observed staff members equally engaged in activities such as cleaning the chicken pen or stables next to residents as this nursing professional explains:

*“And I mean I lay on my knees just as much, maybe even worse. It does encourage them to do everything together. That’s super fun though […] And the feeling like you still belong, I think is especially important, because you may suffer from a disease, but about everything that happened in the past, I don’t judge, because that’s not what I’m here for. You just have to be here and now and are responsible for taking care of yourself. Now and then when we take a break, I show a picture of my children or my grandchildren or a crazy movie or a joke from Facebook to them and then we have a good laugh. They love that, because then we are equal, and I am not their boss, because that is sometimes said: ‘Yes that she is the boss,’ and then I say, ‘A dog has a boss, you don’t have a boss.’”* [Liza, CNA, l. 264–271].

Residents were observed to use their talents and expertise. Several residents participating in the activities had a background in farming and advised staff members on, for example, how to build a fence or how to best catch a chicken when they need medication. In this reciprocal relationship, ‘traditional’ gender roles appear to facilitate the use of the environment. Male residents were observed to see themselves as the persons who have to be of assistance to female care staff as described by a nursing professional:

*“You are then going to put them in a certain role anyway. That you indeed say like, ‘Tom, can help me with that?’ You know like that and that works. With men that works! And in that respect, you often have an advantage as a woman here. Sometimes you don’t, sometimes you do. It’s just the way it is.”* [Liza, CNA, l.466–468].

Hence, the environment enhanced reciprocity in the care relationship as residents care for others instead of being cared for. The observations revealed how residents who gave the impression of being passive and agitated in the living room eagerly engaged in activities for the animals. Verbal reactions and facial expressions indicate joy when animals react positively to them being fed and cared for. As observed in the following field note, a resident with dementia explains how he shared his new role with his daughter.

*“The resident explains how he calls his daughter every morning at 10 a.m. and yesterday he told her that he was going to take care of the animals today. His daughter just really enjoys hearing this he tells proudly. He looks at the ladies [residents] around him and smiles.”* [Fieldnote extract].

#### Theme 3: Seized and missed opportunities for meaningful integration of ADL in the physical green care environment

The third theme demonstrated how the green care environment was used to create a meaningful integration of the environment with resident needs and skills. It is therefore divided into the subtheme: *‘Seized opportunities by meaningful integration’* and ‘*Missed opportunities for resident involvement and integration’*.

Generally, how the green care environment was used by the organization depended on the needs and goals of the residents living in a particular ward. The use of the environment was tailored towards different needs including structure and work character for people with Korsakoff’s disease, a moment of rest for the agitated resident with dementia, or purposeful movement under supervision for the residents with mobility problems.

Seized opportunities for meaningful ADL performance were identified when staff members were mindful of the residents’ needs and skills as well as how the environment contributed to that as the following example illustrates:

*“A staff member asks a group of six residents with dementia sitting around a table outside the animal shelter who wants to cut an apple for the animals. The residents do not respond. A colleague grabs a cutting board, a kitchen knife, a bowl, and an apple. She puts these things in front of a resident and cuts the apple in half. The resident takes an apple in one hand and the knife in the other and begins to cut off pieces. In the hand holding the knife he also holds the cut-off piece of the apple, which the resident then brings to his mouth. This brings the knife close to his mouth, but the activity looks safe and he enjoys his apple considerably.”* [Fieldnote extract].

Hence, the use of the environment was directly linked to ADL performance if staff members saw and seized the opportunities.

Results showed how different staff members took different approaches in using this environment and identifying key strategies. Staff members explained how using the green care environment requires a certain amount of courage or ‘guts’ to experiment with how the environment works for different individual residents. Some staff members indicated how using the environment also results in positive experiences for the residents who never had any interest in nature or animals in the past. Trial and error were identified as a strategy by the staff to maximize the use of the environment especially for residents who struggle to communicate verbally, as this nurse assistant describes:

*“Just trying. Just try it. And if the effect is nothing or you notice that it doesn’t seem to be working, then try a spin on it. Because last week it had gone outside with someone. And they didn’t like it at all. Then I went with her for coffee in the restaurant. And she talked so much. Yes, and then I think, look at that! It does depend. I mean, they can’t say what they want themselves. So you also just have to try to figure out what would be the best thing we could do?”* [Gabrielle, nurse assistant, l. 80–83].

Trial and error as a strategy also implied that staff members take a certain risk with the residents. For instance, the risk of the residents not enjoying the activity or being afraid of the animals on the one hand, and the risk of being exposed to the potential to fall or eat the chicken feed. At the same time, staff members saw how the risk is worth taking in light of the benefits the residents experience from this environment. Staff members described situations in which agitated residents verbally and non-verbally experience joy and fulfillment from these activities or how residents tell them how this environment gives them a purpose. Other staff members observed how sometimes residents appear ‘overstimulated’ or change their mood quickly when they enjoy being in the green care environment.

*‘Missed opportunities for resident involvement and integration’* illustrates, sometimes opportunities for resident involvement in ADLs are missed especially before and after scheduled activities where (un)dressing or washing hands was taken over by staff. These activities seemed not to be seen as part of the animal activity but rather a necessary and quickly performed task. For instance, by using wet wipes for cleaning the hands of the residents, staff members at several observed instances choose convenience over active facilitation of ADL performance:

*“After feeding and petting the animals at the animal enclosure, a staff member discusses with her colleagues we can best wash the residents’ hands. She thinks it is more convenient to do this on the ward because people are cold […]. Inside the living room, the other residents are still in the same places as we found them […]. We stay in the middle of the living room and a moment later, a staff member comes with wet wipes to clean the hands of sir. For each resident who joined us, the care worker wipes their hands.”* [Fieldnote extract].

#### Theme 4: Professional fulfillment and ADL task obligation: views from staff and management

Theme 4 described how the use of the environment was perceived by different staff members in relation to their professional fulfillment and task obligation. This theme is divided into the subthemes ‘*Professional fulfillment by creating shared moments of joy’*, ‘*Task-oriented view on care’* and ‘*Management perspectives on integrating the environment in daily care’*.

As part of the theme professional fulfillment by creating shared moments of joy’, staff members including nursing and activity staff explained how the use of the environment contributes to their professional fulfillment, especially when resident experiences are positive. They described how creating positive and meaningful moments for residents by using this environment makes them feel satisfied when they get home from work. As the following nurse assistant illustrates, staff members enjoy seeing residents happy especially since these moments are sparse and often fade away once the residents return to the inside environment.

*“I feel that, more often than not, it [positive feeling of residents] has receded into the background. That’s only very brief moment of happiness. I just call it happiness, because that what it is. It’s very short and when I’m inside, I often notice that the feeling has faded again. But did the residents enjoy it? I think so. And those small, short moments are very important. That’s what you do it for […]. That was so beautiful! Sometimes, in a moment like that, if they’re happy, then I’m happy too. Then I know, I’ve done well and I think it’s also not just effort or difficult at that moment, but you also gain so much from it!”* [Gabrielle, nurse assistant].

While the common experience of using the environment is positive among the staff members who use it, the subtheme ‘Task-oriented view on care’ illustrates perspectives across all interviewed staff differed on whether the use of the environment feels part of their role and task obligation. Some nursing professionals considered using the environment as part of their job to assist residents in all activities including using the green care environment. Other nursing professionals saw their role in creating ‘small’ meaningful or person-centered moments within the inside ‘traditional ADL environment’ including bath and bedrooms rather than the green care environment. This nursing professional explained that she saw a clear difference between the tasks and responsibilities between those of nursing professionals and those of the activity staff.

*“I think activity staff is responsible for the bigger activities where we sometimes are scheduled to participate in. I think we are responsible for those little extra moments. That one-on-one moment. It doesn’t even have to be very big activities, but yes, a glass of wine or an eggnog, you know is something already. Or indeed doing the nails for the ladies on the ward, which is just something very small because it might only take five minutes […]. Those are just really those little moments already, which is enough for them and I think that’s often forgotten […]. So I think the care staff are a bit more focused on that and activity staff is really more focused on the bigger things.”* [Rose, RN, l.250–258].

Moreover, the quote shows the nurses’ task-oriented view on their role in assisting residents in their daily lives. Staff members differed in perception on whether the use of the environment was viewed as a separate task, or an opportunity to stimulate abilities or brighten the day of the residents appears to limit its potential in practice. Hence, other staff members observed a variety of task obligations among their colleagues. They would see more nursing professionals involved in the activities within the green care environment. With the increased engagement of nursing professionals, they hoped more residents could benefit from this environment. At the same time, activity staff at instances feel left alone with activating and encouraging residents to use green care environment, which has caused some staff members to become disheartened.

The final subtheme ‘*With perspectives on integrat?ing the environment in daily care*’ highlights how ward managers acknowledged the different perceptions and encouraged nursing professionals to seize the opportunities of the green care environment for the benefit of the residents and their own job satisfaction, as stated in one of the interviews:

*“Activity staff are doing this now [activities with animals]. Yes, and I do see them struggling sometimes and they hope that nursing colleagues will pick this up as well. And that just has to do with your team. That’s also what I say: Make your job fun! How much fun is it? Even if you’re a CNA or a registered nurse, you can think I take three residents and I’m going to go to the animals. Come out from behind your computer and also make also fun for yourself to then go with three residents and see them enjoying themselves.”* [Monica, ward manager, l.142–150].

At the same time, staff members and managers acknowledged how the outside environment and especially being in contact with animals is not for everyone. Some staff members were afraid of particular animals or simply preferred to stay inside. Managers saw how optimizing care and the use of the environment required a change in attitude and competences in staff. These changes are especially needed to perform care and environment use in a person-centered way.

The required change is also visible in the way different wards engage in the green care environment and the responsibilities they take on and are able to manage. Results showed how some wards took responsibility in maintaining the green care environment and others did not. An often-mentioned key player for creating shared responsibility in staff across wards and ensuring quality was the social worker of the nursing home. Across wards, managers, direct care workers, and activity staff stressed how the social worker’s enthusiasm and organizing skills led to an increased use of the environment. However, as one of the managers explained, it was their role to ensure continuity in using and sustaining an integrated use of this environment in collaboration with the social worker:

*“Sometimes I do think, that’s also up to me, that I also said to her [the social worker] you should just join a ward meeting again, to tell about it very briefly, even if it’s for 10 minutes, to get that mindset in fellow workers, huh? That’s very often, we have so much to offer […] and sometimes that’s just forgotten in that day-to-day grind. When reminded, staff members think employees it’s ‘oh yes, yes, of course, I can use this or that again’.”* [Monica, ward manager, l.317–321].

## Discussion

This study showed how meaningful opportunities for engaging in ADL performance arise prior, during, and after activities in the green care environment, and how responsibilities like caring for animals motivate engagement and activity. The environment also fosters a reciprocal care relationship between the residents and the staff. However, there are differing opinions among the staff and management on integrating this environment into daily routines. This study was, to the best of our knowledge, the first to explore how the physical green care environment is used to support ADL performance of nursing home residents.

This study illuminated the potential of a green care environment potentially affecting the residents’ abilities of ADL performance. Literature on innovative care environments indicates that opportunities for involvement in activities might be a promising element of maintaining and increasing ADL dependence [20]. Green care environments strongly advocate for resident involvement with meaningfulness as a core mechanism for empowerment [39]. Meaningfulness can be achieved by a purposeful use of the physical environment [40]. The significance of outdoor activities in the green care environment, a coherent integration between these activities, and direct care activities such as ADL creates meaningful opportunities to be explored in further research. Our findings indicate that ADLs are an integrated part of the scheduled activities, within the green care environment throughout various stages (e.g. getting dressed before an activity or washing hands afterward. However, they tend to be overlooked. Direct hands-on ADL care seems to become less prominent in a green care setting as the focus shifts from an indoor, care-centric setting to a more outdoor, productivity-oriented one. As an illustration, tasks like feeding the animals take center stage, while activities related to mobility or handwashing tend to recede into the background and may be at risk of being unnoticed. While this transition is not necessarily negative, this situation could potentially lead to residents missing out on opportunities to actively engage and maintain their functional ADL abilities within a unique and innovative setting.

Considering the potential benefits a green care environment and its animals might have on the residents, more and more nursing homes have integrated them in their facilities [19, 41]. This study shows how staff members play an important role in seizing opportunities within the physical green care environment to facilitate ADL. When integrating fundamental elements of nursing care such as ADLs in a specific context, it is crucial to view ADLs not solely as addressing physical necessities (such as eating, toileting, or personal hygiene) but also as recognizing the psychosocial needs of individuals with dementia (e.g. considerations of dignity, involvement, and information) in the provision of nursing care [42]. This entails that more and more nursing homes expand their definition of ‘care’ beyond the fulfillment of physical needs and pay attention to psychosocial elements of care provision [43, 44]. This is in line with previous research in dementia care, equally recognizing the social and organizational environment next to the often more obvious changes in the physical environment [45, 46]. For example, while most interview participants valued the animal shelter and enjoyed cleaning stables together with the residents, they also mentioned that not all colleagues had an affinity with animals. Here, staff members, as part of the social environment, substantially impact the success of the physical environment. As they play a central role in care delivery, their work environment is of crucial importance for the quality of care delivered [47, 48]. Consequently, the changes resulting from an innovation in their work environment have to be recognized as well.

With this comes an often underestimated, changed understanding of the role of staff members, as demonstrated in earlier studies on ‘Shabazim’– the staff members in Green Houses for people with care needs [49–51]. Here, staff members are seen as companions in the daily life of residents, where the laundry, meal planning, and joint coffee breaks are an equally important part of their tasks as the care delivery. Where especially nursing staff members perceive their role as merely task-focused, specifically in ADL nursing [5], the organization might have to adapt routines nudging staff members towards integrating the activities in a desired context or change the workforce– posing difficulties in times of scarcity of qualified personnel. Qualities or competencies of staff members as described by de Boer, Buist [52] aid in integrating activities for residents into daily practice while being able to take multiple responsibilities within and beyond care activities. This shows how organizational decisions to change the physical environment might not be successful without similarly acknowledging the social and also organizational environment of the organization.

### Limitations

This study has certain limitations. First, although informal conversations were held with residents during observations, their perspectives were not specifically explored in more in-depth discussions or interviews. This could potentially have led to an underrepresentation of the residents’ viewpoints as to how they view the use of the environment and their ADL care experience. Second, despite striving for variety in experiences and professions, for instance in selecting staff members for the interviews, it could, of course, have been the case that there are other critical staff members we did not hear. Those who willingly participate in research and enjoy discussing it may hold a more positive outlook compared to those who may be more hesitant to engage in such discussions.

#### Implications for further research

Given the specific focus of this study, it is recommended to explore more comprehensive qualitative ethnographic research. Further research should concentrate on various types of outdoor environments, extending the duration beyond specific animal-related activities to encompass morning care and other ADL moments throughout the day. Although some demographic characteristics were considered in the current study, this could be enhanced in future studies. Hence, certain biographical data might impact the role an environment has on an individual. For example, aspects such as having an agricultural background, or having an affinity with animals and the outdoors might facilitate the impact of the environment on ADL functioning. This is especially the case when considering the impact of the role of an outdoor environment. Moreover, it would be valuable to conduct systematic experimental research to determine whether utilizing the environment actually improves ADL-functioning over time.

## Conclusions

Our findings suggest that the physical green care environment carries the potential to increase ADL performance. We found that activities within this environment increase opportunities for ADL performance and care before, during, and after activities. Moreover, using this green care environment can motivate the residents to engage in purposeful activities and increase reciprocity in staff-resident relationships. However, there are differing opinions among staff and management on its integration into the residents’ lives and care.

### Electronic supplementary material

Below is the link to the electronic supplementary material.

Supplementary Material 1

## Data Availability

The datasets used and/or analyzed during the current study available from the corresponding author on reasonable request.
