# Supplementary material for: Utilizing the physical green care environment to support activities of daily living for nursing home residents: a focused ethnographic case study
Source: BMC Nurs. 2024 Mar 5;23:160. doi: 10.1186/s12912-024-01782-7 (PMC10913440; doi:10.1186/s12912-024-01782-7)
Supplement: Supplementary file 1 — Supplementary Material 1 [file 12912_2024_1782_MOESM1_ESM.docx]

Appendix 1: Codetree

| Data-extracts | Code examples | Subthemes | Themes |
| --- | --- | --- | --- |
| The activity staff member brings Mr. and Mrs.'s coats, and together we help them put on their coats. Both Mr. and Mrs. are seated in wheelchairs, so we ask both of them to lean forward in order to put on the coats. [Fieldnote extract]  The resident takes off his Birkenstocks and puts socks on his feet. For this, He leans forward in the chair and tries to lift his leg.  Judging by his strained facial expression, this proves to be quite challenging for him, but he manages independently. After putting on both socks, he puts the Birkenstocks back on. I ask if it's not too cold for his feet. He indicates that it's fine and proceeds to put on a hat." [Fieldnote extract] | Getting dressed before going outside | Visibility of ADLs before during and after activities | Theme 1: The (in)visibility of ADL |
| As we walk to the animals, the resident and I and talk about how quiet it is on the ward and how you can see fewer and fewer people through the windows. 'They must all be lying in front of the TV,' he says 'not running around like crazy as we are out here,' he adds with a smile." [Fieldnote extract]  "A resident and I stand two meters away, waiting for the goats to come out. They seem a bit cautious at first but decide to leave their stable. A staff member walks ahead outside, and he follows with the walker to guide the goats outside." [Fieldnote extract] | Being physically active and mobile during activities |  |  |
| Right before the entrance, there is a device with brushes for cleaning shoes. The resident independently uses this to clean his shoes. To do this, he stands on one leg while wiping his shoes on the brushes with the other leg. He keeps one hand on the wall next to the entrance for support. Marco seems to take this task seriously and cleans each shoe on the brushes at least 10 times. [Fieldnote extract] | Cleaning oneself after the activity |  |  |
| You also have something to go to, right? I: and then it's not like, "come, let's go outside." No, but "come, let's go to the animals." That sounds very different. Or then you have a goal, you have a goal, right? A destination. [Interview transcript]  Aren't you going to the gym, swimming...? Indeed, when you work in a structured department, like here in the Korsakov unit, people are used to structure. If we leave people with Korsakov to themselves, they'll stay in bed all day. Simple. Yes, so you try indeed. to stimulate people to do something. [Interview transcript] | Perceived purpose and goals of using the environment | Invisible ADLs |  |
| I am the responsible caregiver for two residents in [this ward], and both of them go to the animals, and spending some time there is also important. Suppose they don't feel like talking—I usually let them be. If they don't feel like it, they don't feel like it. However, I can easily find an entry point by bringing up the animals, the plants, or the birds, and then I can usually start a conversation." [Interview transcript] | Easier entry point for conversations | Strengthening the care relationship | Theme 2: Reciprocal care dynamics: Fostering ADL performance through connection and teamwork |
| And so, I have found a way to take care of her, to take her for a shower. Every time, little by little, and after showering, we would go to the animals together. At some point, she started associating it, so every time I went to take care of her, she would ask if we were going to the deer. [Interview transcript] | The strengthened relationship within and beyond intimate ADL care |  |  |
| They are not unintelligent people; they used to function just fine before. It's not like they've lost all of that," he would say to me. "I wouldn't do that yet," he said, so I responded, "Yes, you're absolutely right! How clever of you; I hadn't even thought about that. [Interview transcript] | Using residents talents and expertise | Reciprocity |  |
| Especially in this setting. That's also something I observe. Residents are often taken care of a lot. And then it's precisely the other way around. [Interview transcript] | Reduced hierarchical structures |  |  |
| A staff member asks if he wants to do it himself or if she should lend a hand. The resident. indicates that she should help. [Fieldnote extract]  A staff member stands next to a resident and places the cutting board, 2 knives, 2 apples, and 1 bowl on the table in front of him, and slices the first apple in half with a kitchen knife. She then addresses him directly and asks him if he would like to cut apples for the animals. He takes an apple in one hand and the knife in the other, starting to cut pieces from it. The piece of apple ends up in the hand holding the knife, which he then brings to his mouth. This brings the knife close to his mouth, but the action appears safe, and he greatly enjoys his apple. [Fieldnote extract] | Integration of environment, purpose, and resident capabilities | Seized opportunities | Theme 3: Seized and missed opportunities for meaningful integration of ADL in the physical green care environment |
| In one corner of the room, three staff members are sitting and having a meeting. Two other staff members are in the kitchen, washing dishes. A staff member hands me a coat and asks me to put it on a female resident while she, along with another colleague, is putting coat on a residents in wheelchairs [Fieldnote extract]  A staff member with moist wipes to clean the residents’ hands. For each resident who has come along, the caregiver cleans their hands with the moist wipes. [Fieldnote extract] | Not involving residents in activities, taking over | Missed opportunities |  |
| What do I think about it, hmm... what it means to me... well, that's what it's all about, you know. When you see them coming in from their home situation, not wanting or accepting anything, and then, with very small steps, you can ultimately achieve this, well then I think 'I'm in the right place,' and that's actually the icing on the cake. That's what you do it for. [Interview transcript]  Because, in the end, you're doing it all for... quality time or just for more beautiful moments or something like that.. [Interview transcript]  "Due to workload, but also just, you know, sometimes you just don't order something, so that even something small is actually sufficient. Just that moment, that small moment. And I think that's something we need to become more aware of. It's just nice that an animal enclosure has been added, and you can just see that a resident becomes much happier because of it. And seeing themselves in the photo, last week I showed him the pictures where he's holding the chicken: 'Ah, that's me' (laughs). P: And then it's- that sense of pride, you know, just that moment back, that recognition. And then you see that, the laughter, and you think, that's what you do it for. So, yes, you definitely see the effect of activities on the behavior of residents. And if you can do just one activity and don't have to resort to medication, that's certainly an advantage as well." [Interview transcript] | That’s what you do it for | Professional fulfillment by creating shared moments of joy | Theme 4: Professional fulfillment and ADL task obligation: Views from staff and management |
| And do you see the animal enclosure as something [big or small]? Is it a small addition or is it really something bigger just because it's a bit further away? We talk about those small things and big activities where the activity staff is mainly responsible. Does the animal enclosure fall somewhere in between, or do you say that it's really something big, you don't use it as easily as just painting someone's nails, for example?"  Yeah, it's just here, very black and white thinking, you're activity staff so you're meant for that, that's just how it is. And yes, it's ingrained, and I've advocated for it a lot, but you just can't get it out. And sometimes it's a shame […]it's quickly said, 'Why did you go there? That's not your task, and you were needed here, and I wasn't needed here because there was nothing here.' So, yeah, new people are discouraged, and then they won't do it anymore. And that's so unfortunate. I think, I can damn well do it, you know? There are people who still see it, and then it's dismissed or like, no. I find that unfortunate. [Interview transcript]  How should I say it? It's all so, a bit stuck, can I say that? Stuck in a rut, that you're standing there, and for the rest, you just stay there. And no, that's not going to work. Last week, I really said, listen, I am a care assistant! I say it, the part about individual attention for people is sometimes forgotten here. And that's what I'm here for. […] And that's what appealed to me about the job. To do that, because it's a bit of attention they don't get. To still give that, I find that very important. [Interview transcript] | Role/identity conflict staff members | Task-oriented view on care |  |
| And then, of course, it was like... Okay, you know, you're admitted to the nursing home. Well, then you leave your entire identity and personality outside the door, and you get admitted. Well, that's how we do it here. And then you completely embrace the whole nursing home ideas, the clinical aspect. And now, of course, we look at it very differently. But that requires a shift for employees. We have employees here who have been working for 30 years. And that really demands a turnaround. [Interview transcript] |  | Management perspectives on integrating the environment in daily care |  |
